# Adopting digital technologies and 3D printing in prosthetics and orthotics: Lessons from a decade of clinical practice

**DOI:** 10.33137/cpoj.v9i1.47211

**Published:** 2026-03-11

**Authors:** D Blocka

**Affiliations:** Boundless Biomechanical Bracing, Toronto, ON, Canada.

**Keywords:** 3D Printing, Digital Workflows, Additive Manufacturing, Clinical Practice, Professional Identity, Prosthetics, Orthotics

## Abstract

Over the past decade, digital workflows and 3D printing have shifted from emerging innovations to mainstream considerations within prosthetics and orthotics (P&O). While these technologies promise improved reproducibility, enhanced documentation, and new design possibilities, their sustained integration into clinical practice has revealed both meaningful benefits and significant challenges. Drawing on nearly ten years of clinical adoption within a busy orthotic practice, this professional opinion article reflects on the lived realities of incorporating digital pathways and additive manufacturing into patient care. Areas of durable benefit include improved design consistency, digital traceability, and educational value. However, total cost of ownership, workflow fragility, material limitations at fitting, and evolving workforce expectations present ongoing considerations. Importantly, digital tools have not eliminated clinical craftsmanship but have reshaped where and how it occurs. As artificial intelligence and data-driven systems emerge, the profession faces renewed responsibility to ensure that digital transformation strengthens, rather than dilutes, clinical reasoning and professional identity. Thoughtful, clinician-led adoption remains essential to sustaining patient-centred care in an increasingly digital environment.

Over the past decade, digital adoption and 3D printing have moved from a fringe innovation to a mainstream interest of the prosthetics and orthotics (P&O) profession. For many clinicians, it represents opportunity: increased consistency, digital traceability and the promise of more efficient, personalized care. For others, it remains a source of frustration, cost pressure and unanswered questions about clinical value and professional identity.

Having worked in a busy orthotic clinical practice for nearly four decades and having led the adoption of digital workflows and additive manufacturing within a clinical practice for almost ten years, I have experienced and seen both sides of this transition. This article reflects on what the sustained incorporation of digital pathways and 3D printing has genuinely delivered, where it has fallen short, and what lessons may be useful for practitioners, educators, and decision-makers considering or navigating similar paths.

This is not a technical review, nor an argument for or against digital manufacturing. Rather, it is a reflection on lived experience: what happens when promising technology meets the realities of clinical care against all odds.

## Early Expectations and the Appeal of Digital Manufacturing

The initial appeal of incorporating digital pathways and 3D printing in P&O was compelling. Digital capture, CAD-based modification and automated fabrication promised improved reproducibility, reduced manual labour and new design possibilities not achievable through traditional methods. For many of us, these tools appeared to offer a pathway toward modernizing workflows while addressing longstanding challenges related to consistency, documentation and scalability.

Early adopters were often motivated not by novelty, but by practical considerations: the desire to standardize processes, improve repeatability and reduce variability between practitioners or technicians. There was also a sense that digital methods could help align clinical practice with broader trends in healthcare innovation and manufacturing.

In practice, the journey from promise to impact has proven to be neither linear nor uniform.

## What Has Worked Well

From a clinical operations perspective, some benefits of digital adoption have proven durable.

Digital design workflows have improved consistency in device geometry and record-keeping. Once a design is finalized, it can be revisited, modified or reproduced with far greater precision than traditional plaster-based methods. This has been particularly valuable in follow-up care, bilateral device production and situations where patients return after extended intervals.

Digital files also provide an educational advantage. Students and early-career clinicians can visualize design intent, understand modification logic and compare iterations in ways that are difficult to achieve with manual techniques alone. In this sense, digital tools have enhanced, not replaced, clinical reasoning when integrated thoughtfully into training environments.

Additive manufacturing has also enabled design features that are difficult or inefficient to fabricate manually, including variable thickness, internal lattice structures and controlled flexibility. In selected cases, these capabilities have translated into lighter devices and improved patient comfort.

These gains are real and meaningful. However, they do not tell the whole story.

## The Reality of Cost, Complexity and Workflow Fragility

One of the most persistent misconceptions about digital adoption/3D printing in P&O is that it inherently reduces cost. Total cost of ownership extends well beyond the printer itself. Capital investment, materials, maintenance, service contracts, software licensing, staff training and downtime all factor into the equation.

For clinical practices, these costs are not theoretical. They directly affect sustainability, pricing models and staff workload. High-end industrial printers, while capable of producing robust clinical devices, introduce dependencies on vendors and service ecosystems that are often outside the clinician’s control.

Digital workflows can also be fragile. A failed scan, software incompatibility, corrupted file or printer malfunction can disrupt production in ways that traditional fabrication methods rarely do. While redundancy and hybrid workflows can mitigate some of these risks, they also reduce the efficiency gains that digital systems are meant to provide.

Over time, one realizes that digital manufacturing is not inherently simpler.

## Fit, Adjustment and the Continuation of Hands-On Practice

Perhaps the most under-discussed aspect of digitally driven and 3D printed orthoses is what happens at the point of fitting.

Despite improvements in scanning and design software, no digital model fully captures the dynamic, soft-tissue interactions that occur when a patient stands, walks, or loads an orthosis. As a result, clinical fit remains an iterative process. In addition, the process of hands-off digital scanning differs fundamentally from traditional hands-on casting techniques. During plaster casting, the clinician can influence limb alignment, apply controlled tissue compression, and guide the shape capture process in real time. By contrast, many scanning approaches capture the limb surface passively, reducing the practitioner’s ability to influence how the underlying anatomy and soft tissues are represented in the digital model. This subtle but important difference in shape capture can affect subsequent device design and represents a limitation of purely digital workflows that clinicians must learn to manage.

Printed materials, particularly high-performance polymers, often behave differently under heat and stress than traditional thermoplastics. While they may offer strength and durability, they can be more challenging to adjust chairside. This places additional demands on clinicians, who must adapt traditional fitting skills to new material properties. Recent work exploring orthotists’ experiences with adjusting 3D printed ankle foot orthoses highlights these practical challenges and the evolving nature of chairside modification in digitally manufactured devices.^1^

In practice, 3D printing has not eliminated craftsmanship, it has shifted where and how it occurs. The assumption that digital design alone can replace clinical judgement has proven unrealistic. Fit, comfort and function still depend on the practitioner’s ability to assess, modify and respond to patient feedback in real time.

## Workforce and Profession Implications

As digital tools become more prevalent, they inevitably influence how we define competence within the profession.

Orthotists and prosthetists are increasingly expected to navigate scanning systems, CAD environments and digital workflows alongside traditional clinical skills. This hybrid skillset can be empowering, but it can also be overwhelming, particularly when training pathways and continuing education have not fully adapted.

There is a risk that digital proficiency becomes conflated with clinical expertise. While technical fluency is important, it does not replace foundational knowledge of anatomy, biomechanics and patient interaction. In fact, poorly applied digital tools can obscure clinical reasoning rather than enhance it.

Education programs and professional bodies face the challenge of integrating digital competencies without diminishing the core identity of the profession. The goal should not be to create “technicians of software” but clinicians who can use digital tools critically and appropriately.

## Key Lessons from Sustained Adoption

Looking back, several lessons stand out:

- First, context matters. What works in a large, centralized facility may not translate well to small or resource-limited practices. Digital adoption should be scaled to clinical need, not driven by external expectations.
- Second, hybrid workflows are often more resilient than fully digital ones. Maintaining the ability to revert to traditional methods when digital systems fail is not a step backward, it is a pragmatic safeguard.
- Third, clinician-led strategy is essential. When technology adoption is driven primarily by vendors or external narratives, it often misaligns with clinical priorities. Sustainable integration requires practitioners to define problems first and select tools second.
- Finally, time must be valued realistically. Digital workflows demand significant upfront investment financially, in learning and process redesign. Expecting immediate efficiency gains can lead to disappointment and abandonment.

## Looking Forward: From Tools to Systems

Emerging developments, particularly in artificial intelligence and data-driven design, will likely intensify these conversations. The potential to automate aspects of shape modification, predict fit outcomes or learn from large datasets is intriguing. However, these tools will only be as effective as the clinical frameworks guiding their use.

As a profession, we must remain actively involved in shaping how digital systems evolve. This includes advocating for standards, ethical use of patient data and educational models that preserve clinical reasoning at the center of care.

3D printing should be viewed not as an endpoint, but as one component within a broader digital ecosystem. One that supports, rather than supplants, professional judgement.

## CONCLUSION

After nearly a decade of clinical use, incorporating digital pathways and 3D printing in prosthetics and orthotics can be neither dismissed as hype nor embraced uncritically as a solution. It is a powerful set of tools with genuine benefits and equally real limitations.

The challenge before us is not whether to adopt digital manufacturing, but how to do so thoughtfully, sustainably and in a manner that strengthens the profession. This requires reflection, shared experience and an ongoing willingness to question assumptions, especially our own.

If the adoption of digital pathways and 3D printing is to fulfill its promise in Prosthetics and Orthotics, it must remain firmly grounded in the realities of patient care and the expertise of those who deliver it.

## AUTHOR BIOGRAPHY

**Daniel Blocka**, B.Sc., C.O.(c), FISPO, F.C.B.C.

Since 1988, Dan has been the President and owner of Boundless Biomechanical Bracing Inc., a large clinical service provider in Orthotics in the Toronto area. Dan Blocka is a Past President of the International Society for Prosthetics & Orthotics (ISPO) serving the term as President from 2007 to 2010. Dan also served as an ISPO Education Committee chair and member in vary capacities from 1998 to 2019. He currently serves on the Global Educators Meeting (GEM) Working Group. In March 2018, Dan was awarded ISPO Fellowship. Dan has been on the Board of Directors of Exceed Worldwide since 2011 & also has served on Board of Directors of Human Study e.V. since 2018. Throughout an almost 40-year span, Dan was a Professor and Coordinator of the George Brown College Prosthetic and Orthotic Educational Programs in Toronto.
